# Curie-Weiss behavior of liquid structure and ideal glass state

**DOI:** 10.1038/s41598-019-54758-y

**Published:** 2019-12-09

**Authors:** C. W. Ryu, W. Dmowski, K. F. Kelton, G. W. Lee, E. S. Park, J. R. Morris, T. Egami

**Affiliations:** 1 Department of Materials Science and Engineering, University of Tennessee, Knoxville, TN 37996 USA; 2 Research Institute of Advanced Materials, Department of Materials Science and Engineering, Seoul National University, Seoul, 08826 Republic of Korea; 3 Department of Physics and Institute of Materials Science and Engineering, Washington University, St. Louis, MO 63130 USA; 4 Korea Research Institute of Standards and Science, Daejon, 34113 Republic of Korea; 5 Department of Nano Science, University of Science and Technology, Daejon, 34113 Republic of Korea; 6 Oak Ridge National Laboratory, Oak Ridge, TN 37831 USA; 7 Ames Laboratory, Ames, IA 50011 USA; 8 Department of Physics and Astronomy, University of Tennessee, Knoxville, TN 37996 USA

**Keywords:** Structure of solids and liquids, Atomistic models

## Abstract

We present the results of a structural study of metallic alloy liquids from high temperature through the glass transition. We use high energy X-ray scattering and electro-static levitation in combination with molecular dynamics simulation and show that the height of the first peak of the structure function, *S*(*Q*) − 1, follows the Curie-Weiss law. The structural coherence length is proportional to the height of the first peak, and we suggest that its increase with cooling may be related to the rapid increase in viscosity. The Curie temperature is negative, implying an analogy with spin-glass. The Curie-Weiss behavior provides a pathway to an ideal glass state, a state with long-range correlation without lattice periodicity, which is characterized by highly diverse local structures, reminiscent of spin-glass.

## Introduction

When a liquid is supercooled by avoiding crystallization with fast cooling or with reduced heterogeneous nucleation^1^, the viscosity increases rapidly with cooling and a liquid becomes a glass^2,3^. How and why a liquid changes into a glass has not been fully answered even today^4,5^. In order to provide an answer to this question it is imperative to know how the structure of supercooled liquid changes with temperature. However, this is not an easy task, because simple liquids crystallize quickly out of the supercooled state, and stable glass-forming liquids, such as some organic liquids, are complex in structure, making it difficult to characterize their relevant structural features.

The purpose of this work is to study the temperature-dependent structure of the supercooled metallic liquid by experiment and simulation. Experimentally, we determine the structure of a relatively stable metallic alloy liquid in the supercooled state by high-energy X-ray diffraction using electro-static levitation^6^. This method allows studying diffraction from a liquid without contact with a container, thus stabilizing the supercooled liquid by avoiding nucleation of crystals. At the same time, we carry out molecular dynamics (MD) simulations on metallic liquids with various compositions.

## Results and Discussion

### Structure function

The structure function, *S*(*Q*), where *Q* is the momentum transfer in diffraction, was determined for Pd_42.5_Ni_7.5_Cu_30_P_20_ liquid by high-energy X-ray diffraction using electrostatic levitation over a wide temperature range, from 1100 K through the glass transition temperature, *T*_g_ (=573 K)^7^, and down to 420 K. Measurements at higher temperatures are prevented by sample evaporation. The Pd_42.5_Ni_7.5_Cu_30_P_20_ glass is known to be the most stable metallic glass to date^7^. Details of the measurement are describe in the Method section.

**Figure 1:**
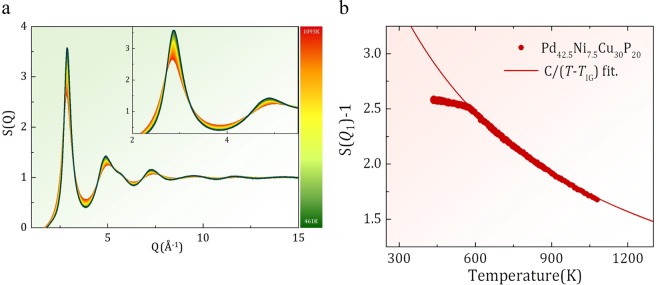
(**a**) The structure function, *S*(*Q*), of Pd_42.5_Ni_7.5_Cu_30_P_20_ liquid at various temperatures determined by high energy x-ray diffraction with electrostatic levitation, (**b**) the variation of the height of the first peak of *S*(*Q*), *S*(*Q*_1_) − 1, of Pd_42.5_Ni_7.5_Cu_30_P_20_ liquid with temperature. The curve is the fit by the Curie-Weiss law with *T*_*IG*_ = −454 K.

Figure 1(a) shows how *S*(*Q*) varies with temperature. As the temperature is lowered the first peak height increases and the peak position shifts slightly outward. The fast-growing first peak indicates that the liquid is trying to establish an order with *Q* at the peak position *Q*_1_ ( = 2.868 Å^−1^ at *T*_*g*_). In the infinite temperature limit, structural order is expected to disappear and *S*(*Q*) = 1 for all *Q*, whereas when a crystal with long-range-order is formed, a peak in *S*(*Q*) diverges to become a Bragg peak. Therefore $\tilde{S}({Q}_{1},T)=S({Q}_{1},T)-1$ can be considered as an “order parameter” of the structure, which changes from zero for total disorder to infinity for long-range order^8^. Interestingly, we found that $\tilde{S}({Q}_{1},T)$ follows the Curie-Weiss law widely found for magnetic materials,

1 $$\tilde{S}({Q}_{1},T)=\frac{C}{T-{T}_{IG}},$$

above *T*_*g*_ with a negative value of the Curie temperature, *T*_*IG*_ = −454 K, as shown in Fig. 1(b). As discussed below *T*_*IG*_ is the temperature at which the ideal glass state is reached in extrapolation. The data deviates sharply from this law below *T*_*g*_ and *S*(*Q*_1_, *T*) shows slower variation. In the liquid state the structure varies with temperature, becoming more ordered as temperature is lowered, and this variation is the main source of the Curie-Weiss behavior. However, once a liquid becomes a glass, the structure is frozen and does not change any more. The small changes in *S*(*Q*_1_, *T*) below *T*_*g*_ are due to atomic vibrations, *i*.*e*. phonons, just as in a crystalline material.

The Curie-Weiss behavior is widely observed for the magnetic susceptibility of magnetic materials; $\chi (T)={C}_{p}/(T-{\theta }_{p})$, where *θ*_*p*_ is the paramagnetic Curie temperature. The divergence of $\tilde{S}({Q}_{1},T)$ would be associated with the development of long-range correlations of density fluctuations, in the same manner as the diverging susceptibility *χ* is associated with the development of long-range magnetic order. The implications of the value of *T*_*IG*_ being negative are discussed below.

**Figure 2:**
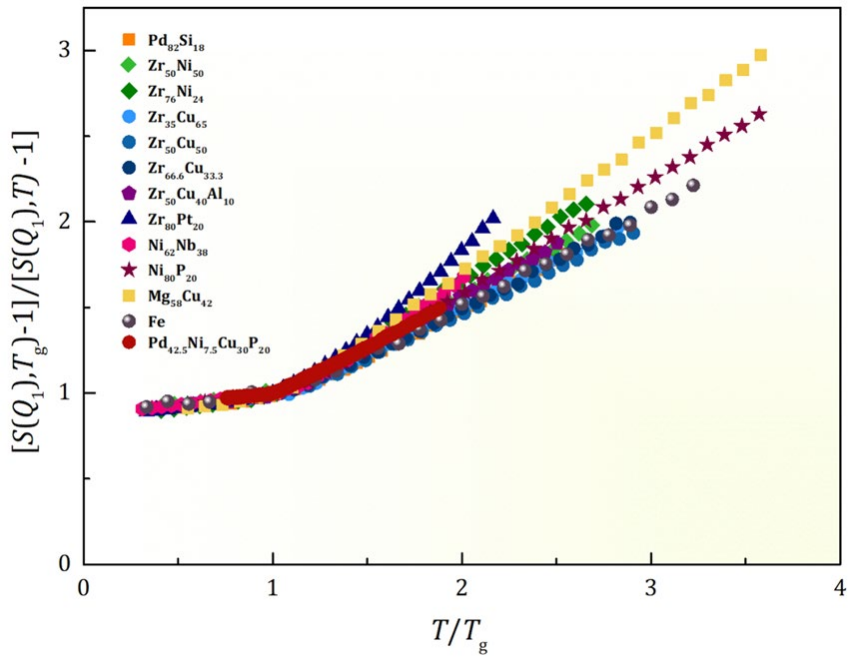
The plot of 1/[*S*(*Q*_1_) − 1] for various alloy liquids by molecular dynamics simulation, and the experimental result for Pd_42.5_Ni_7.5_Cu_30_P_20_, normalized by the values at *T*_*g*_.

We then tested the generality of this result by carrying out molecular dynamics (MD) simulation for a number of metallic alloys. Details are provided in the Method section and the Supplementary Material (SM). The plots of $1/\tilde{S}({Q}_{1},T)$ for various liquid alloys and liquid Fe are shown in Fig. 2, normalized to the values at *T*_*g*_, including the experimental result for Pd_42.5_Ni_7.5_Cu_30_P_20_. The values of $1/\tilde{S}({Q}_{1},T)$ are nearly linear with temperature, indicating that indeed the Curie-Weiss law is valid for all compositions considered here, supporting the view that this law applies generally to metallic liquid alloys. The Curie-Weiss analysis on the second peak is discussed in the SM. A slightly different behavior was reported in an earlier experimental study of Zr_60_Cu_30_Al_10_ liquid^9^. However, the same study found significant changes in the chemical short-range order (CSRO) with temperature, and the outcome was most likely affected by the temperature-dependent CSRO.

The structure of liquid or glass can be conveniently described by the atomic pair-distribution function (PDF), *g*(*r*) (see SM). Classic work by Ornstein and Zernike^10^ predicts that the long-range part of *g*(*r*) decays with *r* as $\exp (\,-\,r/{\xi }_{s}(T))/r$, where *ξ*_*s*_(*T*) is the structural coherence length. Indeed for Pd_42.5_Ni_7.5_Cu_30_P_20_ liquid the long-range part of the reduced PDF, $G(r)=4\pi r{\rho }_{0}[g(r)-1]$, where *ρ*_0_ is the atomic number density, shows an exponential behavior (Fig. S3) with a slope which varies with temperature. Therefore we may write,

2 $$G(r)={G}_{0}(r)\,\exp \,(-\,\frac{r}{{\xi }_{s}(T)}),$$

where *G*_0_(r) is the PDF of an ideal liquid, or more likely an ideal glass in which *ξ*_*s*_ diverges. Now *S*(*Q*) is obtained by the Fourier-transformation of *g*(*r*). Because the Fourier-transformation of Eq. (2) is a Lorentzian function, *S*(*Q*) is a convolution of *S*_0_(*Q*), the structure function of the ideal structure, by the Lorentzian peak shape for *S*(*Q*),

3 $$S(Q)-1=\int [{S}_{0}(Q^{\prime} )-1]{P}_{Q}(Q,Q^{\prime} )\,dQ^{\prime} ,$$

where *P*_*Q*_ is the Lorentzian broadening function,

4 $${P}_{Q}(Q,Q^{\prime} )=\frac{{\xi }_{s}/\pi }{{\xi }_{s}^{2}{(Q-Q^{\prime} )}^{2}+1}.$$

Indeed, the first peak of *S*(*Q*) is fit quite well by the Lorentzian function as shown in Fig. S4 (SM), because the primary contributions to the first peak of *S*(*Q*) come from the long-range part of *g*(*r*)^11^. Therefore, *S*(*Q*_1_) − 1 ∝ *ξ*_*s*_(*T*), and *ξ*_*s*_(*T*) ∝ 1/(*T* − *T*_*IG*_), which diverges at *T*_*IG*_.

**Figure 3:**
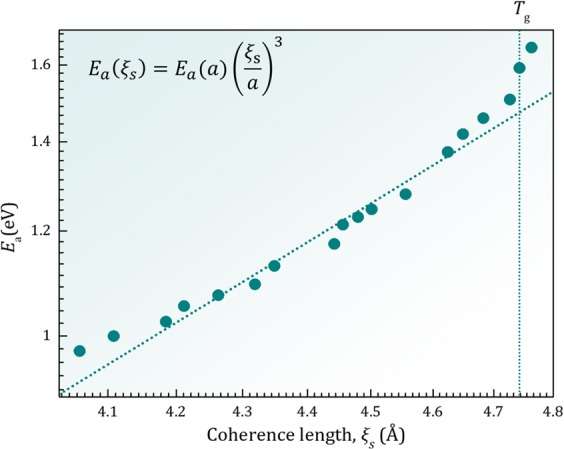
The apparent activation energy for viscosity, *E*_*a*_(*T*), plotted against the structural coherence length, *ξ*_s_(*T*), in log-log scale for Pd_42.5_Ni_7.5_Cu_30_P_20_ liquid above *T*_*g*_. The dotted line indicates the slope of 3. Data on *ξ*_s_(*T*) below *T*_*g*_ are out of equilibrium due to the high rate of temperature scan for the x-ray scattering measurement.

Interestingly, the slope above *T*_*g*_ in Fig. 2,

5 $${m}_{s}=\frac{d}{d(T/{T}_{g})}(\frac{\tilde{S}({Q}_{1},{T}_{g})}{\tilde{S}({Q}_{1},T)}),$$

was found to be directly related to the fragility^2^ defined as

6 $$m={\frac{d\log \eta (T)}{d({T}_{g}/T)}|}_{T={T}_{g}},$$

where *η*(*T*) is temperature dependent viscosity, by $m\propto {m}_{s}^{3.6}$, as shown in Fig. S5. Thus fragility, the rate of change with temperature for viscosity, is related to that for the structure, as suggested earlier^12^. Because $\mathop{S}\limits^{ \sim }({Q}_{1},T)\propto {\xi }_{s}(T)$ if we write $\eta (T)={\eta }_{\infty }exp({E}_{a}(T)/{k}_{B}T)$, this result suggests that the activation energy, *E*_*a*_, varies with *ξ*_s_ as ${E}_{a}\propto {\xi }_{s}^{d}$ with *d* = 3.6. By plotting ${E}_{a}(T)={k}_{B}T\,ln(\eta /{\eta }_{\infty })$ against *ξ*_*S*_(*T*) for Pd_42.5_Ni_7.5_Cu_30_P_20_ liquid with the experimental values of viscosity^13^ we can directly assess how *E*_*a*_(*T*) varies with *ξ*_s_(*T*) (See SM for the value of *η*_∞_). Such a plot given in Fig. 3 shows that just above *T*_*g*_,

7 $${E}_{a}({\xi }_{s})={E}_{a}(a){(\frac{{\xi }_{s}}{a})}^{d}$$

where *a* is the nearest neighbor distance, with *d* = 3 and *E*_*a*_(*a*) = 0.30 eV. The value of *E*_*a*_(*a*) is close to those for high-temperature liquids and corresponds to the energy of cutting one atomic bond^14^. Equation (7) suggests that it is possible that the increased structural coherence volume with cooling directly affects the activation energy leading to the rapid increase in viscosity toward the glass transition.

### Ideal glass state

The extrapolated divergence of viscosity^15^ and the configurational entropy catastrophe^16^ below *T*_*g*_ prompted researchers to speculate that the origin of glass formation is the frustrated local structural order; the increased structural order toward an ideal glass state at a temperature below *T*_*g*_ causes kinetic slow-down, but the structural order is frustrated and never becomes long-range^17–22^. A well-known example of frustrated order is the icosahedral order^17–19,22^. Several theories predict the ideal glass state to exist below *T*_*g*_, characterized either by structural coherence or by complex high-order correlations^21,23^.

The result presented here suggests a different scenario than those proposed by the existing theories. We predict that the viscosity divergence occurs not just below *T*_*g*_ but at a negative temperature. We should note that the prediction of viscosity divergence strongly depends on the model. Even though the most widely used Vogel-Fulcher-Tammann (VFT) model^15^ predict divergence below *T*_*g*_, this model shows poor fit to the data for liquid metal alloys, and other models which show better fit predict divergence only at *T* = 0 or *T* → −∞^24^. Therefore our prediction of viscosity divergence at a negative temperature is not out of line compared to other models. In terms of analogy to magnetism the existing ideas assume a positive Curie temperature, whereas our observation indicates a negative Curie temperature, implying a negative effective exchange constant *J* for pseudo-spins for structure. Indeed, in the pseudo-spin model of local shear fluctuations in liquid *J* is negative^25^ (see SM). A negative *J* in a disordered liquid structure should result in the spin-glass state at low temperature, because the preponderance of triangles and tetrahedra in the structure leads to spin frustration, suggesting close similarities in the behavior between metallic liquid and spin-glass^23^.

**Figure 4:**
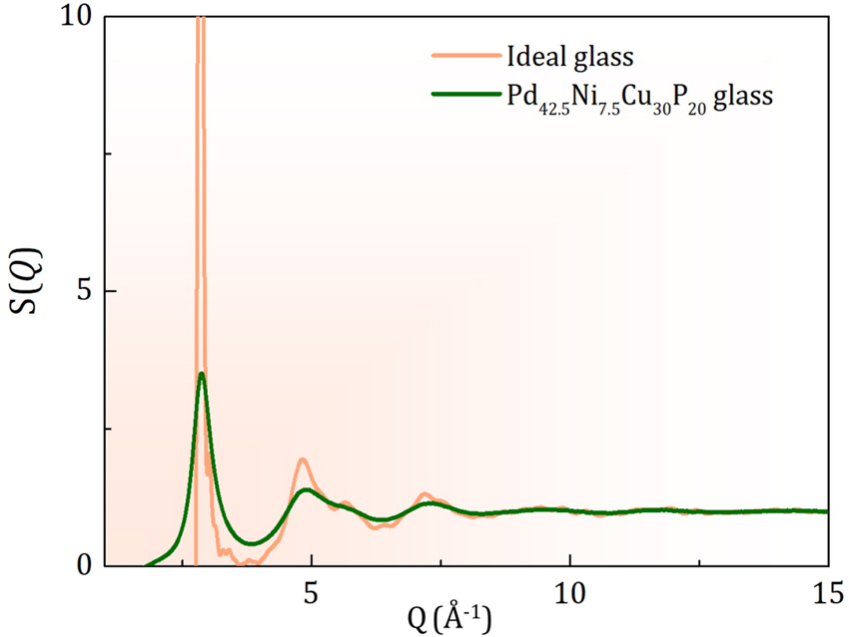
*S*(*Q*) for the ideal glass obtained by the Fourier-transformation of *G*_0_(*r*). The height of the first peak depends on the termination in *Q* space. Ideally it should be a *δ*-function.

**Figure 5:**
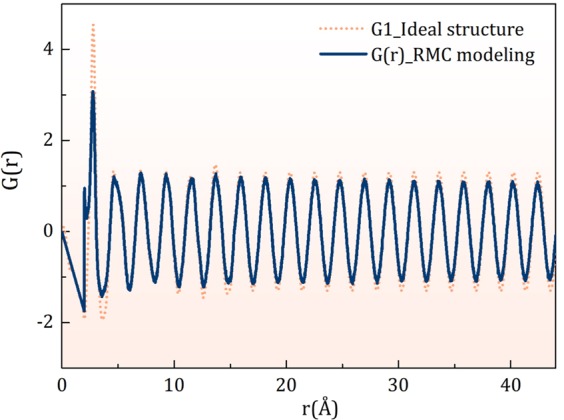
The *G*(*r*) of the structure obtained by the reverse Monte-Carlo method to model the ideal structure, compared to *G*_1_(*r*), the *G*(*r*) for Pd_42.5_Ni_7.5_Cu_30_P_20_ at 600 K modified by multiplying through exp((*r* − *r*_*c*_)/*ξ*_*s*_) for *r* > *r*_*c*_, with *r*_*c*_ = 3.67 Å and extending it to large *r*. A small subpeak of the first peak of *G*(*r*) was caused by the constraint of the minimum distance at 2 Å.

The Eq. (2) allows to predict *G*_0_(*r*) by multiplying *G*(*r*) through exp(*r/ξ*_*s*_), as shown in Fig. S6 (SM) for Pd_42.5_Ni_7.5_Cu_30_P_20_ at 600 K. The long-range part of *G*_0_(*r*) beyond 6 Å approximately is given by *A*sin(*Q*_1_*r* + *δ*). It is interesting to note that the *G*(*r*) for a crystal maintains irregular oscillations with similar amplitudes as *r* → ∞^26^. Therefore *G*_0_(*r*) having a constant amplitude and the damping of *G*_0_(*r*) in liquid by exp(−*r/ξ*_*s*_) are physically reasonable. The corresponding *S*_0_(*Q*), calculated from *G*_0_(*r*), is dominated by the Bragg-like first peak as shown in Fig. 4. This result leads us to a new concept of the ideal glass state; a structure with long-range correlation without lattice periodicity. The quasicrystal was the first example of such a state with two incommensurate periodicities^27^. For the ideal glass state, the structure is characterized not only by two periodicity vectors as in quasicrystal, but by an infinite number of periodicity vectors, ***Q***_**1**_, of which length is fixed but direction continuously covers all the 4*π* solid angle, forming a Bragg sphere. Thus, unlike a quasicrystal, this state has no orientational order. A real-space example of such a structure, determined by the reverse Monte-Carlo (RMC) method^28^ by trying to reproduce this ideal *S*_0_(*Q*), is shown in Fig. 5 in terms of *G*(*r*) with long-range oscillation as discussed in SM. The *G*(*r*) of the model has a fairly wide first peak, and the Voronoi analysis^29^ of the model, presented in SM, shows a very wide distribution of the local structures, with many local polyhedra having the probability of ~1%. Therefore the increase in structural coherence, such as the one indicated by the increase in *ξ*_s_(*T*), does not require domination by any particular local motifs, such as an icosahedron. The medium-range structural coherence does not require coherence in the atomic structure. It only implies coherence in collective density waves.

We suggest that the divergent tendency of icosahedral correlation below *T*_*g*_ observed in simple systems, such as a one-component liquid^17–19^, is actually caused by a deviation from the ideal structure to a local crystalline or quasicrystalline state dominated by icosahedral local structure, rather than an approach toward the ideal glass state. In the ideal liquid structure proposed here the long-range structural coherence is established at the expense of local order which remains ill-defined; a case of order out of disorder. Indeed as we pointed out the negative Curie temperature suggests a spin-glass-like state, which is characterized by high diversity of local spin configurations^30^. In such systems freezing should occur by local trapping of an atom to a cage because of the discrete nature of coordination, sufficiently explaining the glass transition^31^. The structural coherence *ξ*_s_(*T*) must relate to the strength of the cage as implied by Eq. (7).

## Conclusion

Our result suggests that the rapid increase in viscosity of liquid upon cooling is caused by increasing structural medium-range order toward an ideal glass. The structurally coherent ideal glass obtained by extrapolation to *T*_*IG*_ is characterized by high diversity in local structures, and is not dominated by a particular motifs, such as an icosahedron. By adjusting chemical composition it may be possible to create a glass which is close to the structurally coherent ideal glass state predicted here. To create such a glass we may need a large number of elements with different atomic sizes to be mixed in order to create highly diverse atomic environments. The Fourier-transform of the potential energy, *ϕ*(*Q*), should have a deep minimum at *Q*_1_, for instance by satisfying *Q*_1_ = 2*k*_*F*_, where *k*_*F*_ is the Fermi momentum^32^. It is possible that such a structure has unusual properties, such as high stability and high mechanical strength, just as the recently developed ultra-stable glasses^33,34^. In this report we presented only the results of experiment and simulation, but the origin of the Curie-Weiss law can be elucidated in terms of the atomic-level shear strain fluctuations^35^, as discussed briefly in SM and described in more detail elsewhere^36^.

## Methods

### X-ray diffraction

The high-energy X-ray diffraction measurements were carried out at the 6-ID-D beamline of the Advanced Photon Source (APS), Argonne National Laboratory, with an incident X-ray energy of 131 keV in a transmission geometry with a 2D detector. The samples (50–80 mg) were electrostatically levitated and heated by laser using the Washington University Beamline Electrostatic Levitation (WU-BESL) facility^6^ to determine the structure function *S*(*Q*) as a function of temperature during continuous cooling from 1100 K to 420 K. The *Q* resolution (FWHM) was 0.06 Å^−1^, and the cooling rate was ~5 K/s at the beginning and ~0.5 K/s at the end. The 2D diffraction data were collected with the rate of 1 frame per second.

### Simulation methods

MD simulations were carried out using the LAMMPS software^37^ for the systems with 16000 or 32000 atoms. We employed the embedded atom method (EAM) potentials for alloys^38–45^ as shown in Table S1 and the modified Johnson potential for iron^46^. The sample was melted at 2000K under the NPT assemble. For each temperature, the sample was equilibrated for 1 *ns* and the temperature was gradually decreased by 50 K in each step. The structure function, *S*(*Q*), was calculated without weighing factor, with each atom contributing with the same scattering strength.

The reverse Monte-Carlo simulation^47^ was carried out to produce the atomistic model for the ideal state. To determine the coordination number (CN) and to characterize the local atomic environment, we used the Voronoi tessellation method. The OVITO software package^48^ was used to construct the Voronoi polyhedra. To eliminate very small Voronoi faces due to the second neighbors the minimum Voronoi area of 2% (of the whole surface) was imposed.

## Supplementary information

Supplementary information

Supplementary information

## Data Availability

The data acquired for this study (data for Figs. 1–5 and S1–S10, and Tables S1 and S2 in SM) are included in the Supplementary Data Files.
