# Supplementary material for: Curie-Weiss behavior of liquid structure and ideal glass state
Source: Sci Rep. 2019 Dec 9;9:18579. doi: 10.1038/s41598-019-54758-y (PMC6901545; doi:10.1038/s41598-019-54758-y)
Supplement: Supplementary file 1 — Supplementary information [file 41598_2019_54758_MOESM1_ESM.pdf]

Supplementary Material
for "Curie-Weiss behavior of liquid structure and ideal glass state"

C. W. Ryu, W. Dmowski, K. F. Kelton, G. W. Lee,
E. S. Park, J. R. Morris and T. Egami

1. Molecular dynamics simulation

Table S1 shows the list of alloy liquids studied by MD simulations with the reference for the potentials.

Table S1. Metallic alloy liquid models created by MD simulation for analysis in this work.

Composition	***# of atoms***	***EAM potential***
$Pd_{82}Si_{18}$	32000	[38]
$Zr_{50}Ni_{50}$	32000	[39]
$Zr_{76}Ni_{24}$	32000	[39]
$Zr_{35}Cu_{65}$	16000	[40]
$Zr_{50}Cu_{50}$	16000	[40]
$Zr_{66.6}Cu_{33.3}$	16000	[40]
$Zr_{80}Pt_{20}$	32000	[41]
$Zr_{50}Cu_{40}Al_{10}$	32000	[42]
$Ni_{62}Nb_{38}$	16000	[43]
$Ni_{80}P_{20}$	32000	[44]
$Mg_{58}Cu_{42}$	16000	[45]
Fe	16000	[46]

2. Data on $S(Q)$

The plot of $1/[S(Q_1)-1]$ against temperature is shown in Fig. S1 for various alloys. The potentials used for simulation are listed in Method section. The values of T_{IG} and T_g are listed in Table S2. The value of $S(Q_1)$ varies significantly among the alloys.

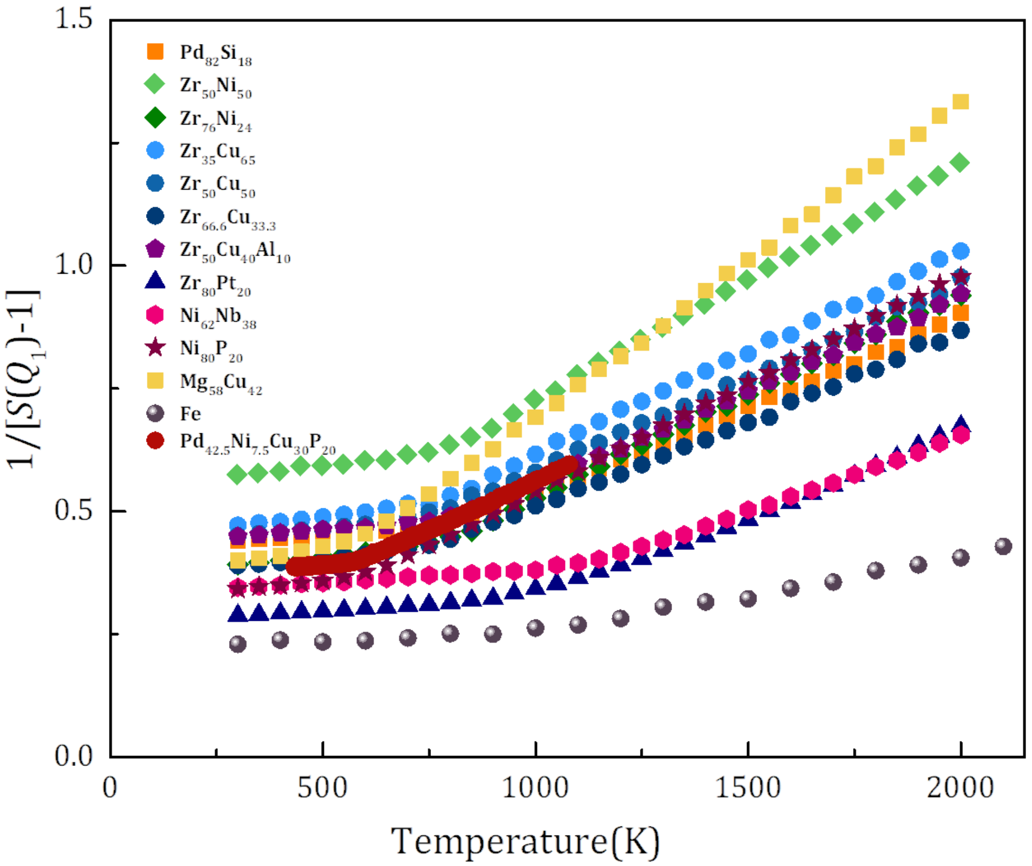

Fig. S1 The plot of $1/[S(Q_1)-1]$ vs. temperature for various alloys. The results other than $Pd_{42.5}Ni_{7.5}Cu_{30}P_{20}$ are by MD simulation.

The similar analysis for the second peak is shown in Fig. S2. The values of T_{IG} for these data, T_{IG2}, are listed also in Table S2, together with the values of T_{IG}/T_g and T_{IG2}/T_g. For many alloy liquids T_{IG2} is negative. However, for Fe T_{IG2} is positive. For liquid Fe $Q_2/Q_1 = 1.71$, very close to the ratio for the icosahedral order [49]. Therefore the growth of the second peak may indicate the development of local icosahedral order [22 – 24].

Liquid of a single-component system or a system with weak dispersion in size tends to form local icosahedral clusters [17 – 22]. In our view they are crystalline or quasicrystalline domains precipitated from the liquid, not part of the ideal liquid. In a single component system liquid is unstable, and upon supercooling crystal or quasicrystal tends to precipitate. Some alloys, such as ZrPt and NiNb, also behave the same way, suggesting they are also dominated by a small number of local structures just as the single-component system. Further details will be discussed elsewhere.

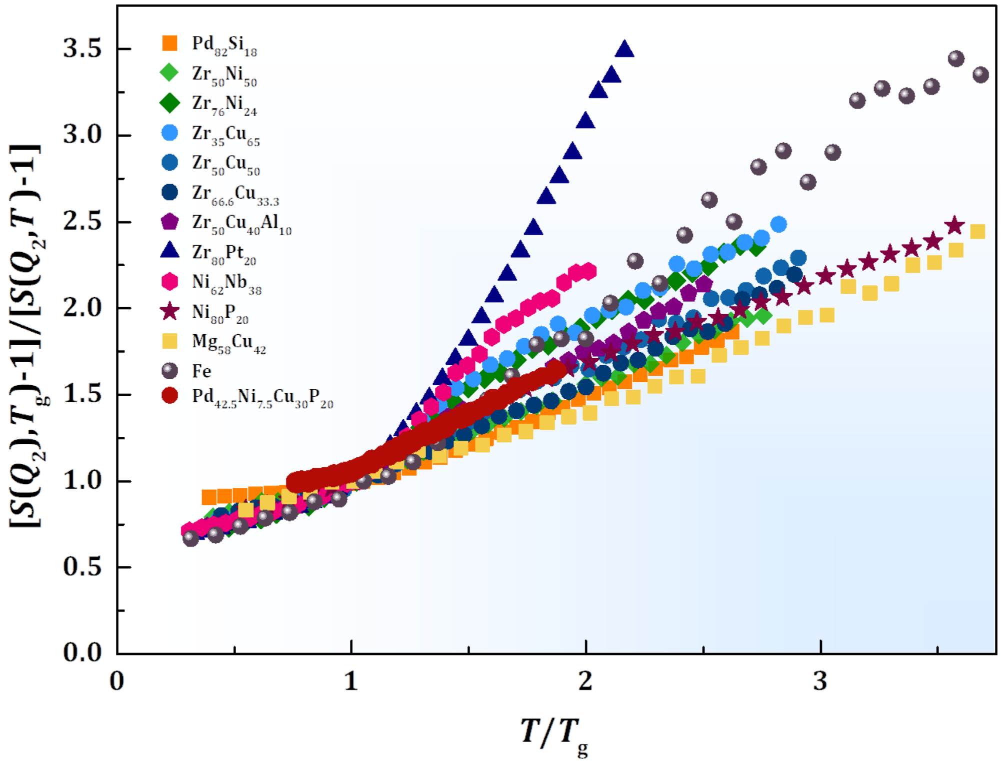

Fig. S2 The plot of $1/[S(Q_2)-1]$ vs. temperature for various alloys, where Q_2 is the position of the second peak in $S(Q)$. The results other than $Pd_{42.5}Ni_{7.5}Cu_{30}P_{20}$ are by MD simulation.

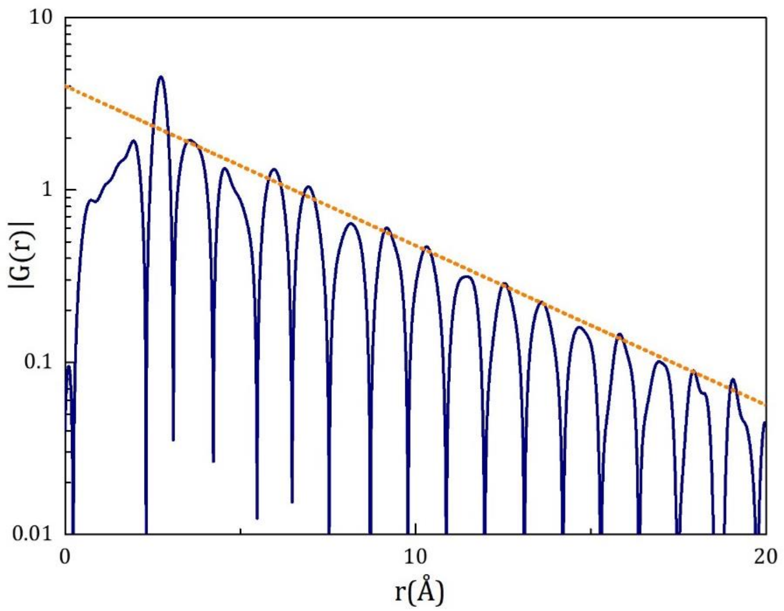

Fig. S3 Exponential decay of $|G(r)|$ for liquid $Pd_{42.5}Ni_{7.5}Cu_{30}P_{20}$ at 600 K. The dashed line is a linear guide to the eye.

The Curie-Weiss law does not appear to apply to complex organic liquids. For instance the height of the first peak of $S(Q)$ for propylene glycol increases with increasing temperature [50]. It is most likely that in organic liquids cooling enhances frustration between packing and local structure favored by covalent bonds, resulting in reduced structural coherence.

3. *Summary of results*

Values of T_g, T_{IG} and T_{IG2}, and their ratios to T_g for various metallic glasses are given in Table S2. The values of T_{IG} were calculated by the fitting of $S(Q_1) - 1$ versus temperature with the $C/(T - T_{IG})$ fit. The glass transition temperature, T_g, was determined from the break in the $1/[S(Q_1)-1]$ plot. The characteristic temperatures for $Pd_{42.5}Ni_{7.5}Cu_{30}P_{20}$ are derived from experiments and the others are results from MD simulations.

Table S2. Values of T_g, T_{IG} and T_{IG2}, and their ratios to T_g for various metallic glasses.

Alloy	**T_g (K)**	**$-T_{IG}$(K)**	**$-T_{IG2}$(K)**	**$-T_{IG1}/T_g$**	**$-T_{IG2}/T_g$**	***Fitting range* (K)**
$Pd_{42.5}Ni_{7.5}Cu_{30}P_{20}$	573	454	326	0.792	0.569	600-1080
$Pd_{82}Si_{18}$	762	661	493	0.867	0.647	800-1600
$Zr_{50}Ni_{50}$	724	518	541	0.715	0.747	750-1350
$Zr_{76}Ni_{24}$	734	341	7	0.465	0.010	700-1600
$Zr_{35}Cu_{65}$	691	525	46	0.760	0.067	700-1800
$Zr_{50}Cu_{50}$	671	601	243	0.896	0.362	700-1800
$Zr_{66.6}Cu_{33.3}$	675	541	422	0.801	0.625	700-1800
$Zr_{50}Cu_{40}Al_{10}$	779	505	257	0.648	0.330	800-1800
$Zr_{80}Pt_{20}$	901	289	-395	0.321	-0.438	900-1600
$Ni_{62}Nb_{38}$	970	435	-327	0.448	-0.337	1000-1800
$Ni_{80}P_{20}$	546	299	343	0.548	0.628	550-1600
$Mg_{58}Cu_{42}$	545	186	893	0.341	1.639	550-1200
Fe	950	737	-132	0.766	-0.139	1100-2000

4. *Atomic pair-distribution function*

The atomic pair-distribution function (PDF) is defined as

$$g(r) = \frac{1}{4\pi r^2 N\rho_0}\sum_{i,j}\left\langle \delta\left(r - r_{ij}\right)\right\rangle, \quad r_{ij} = \left|\mathbf{r}_i - \mathbf{r}_j\right| , \qquad \text{(S1)}$$

where $\mathbf{r}_i$ is the position of the i-th atom, $i = 1, \ldots, N$, $\delta(r)$ is the δ-function, ρ_0 is the atomic number density, and <….> denotes thermal average. It is related to $S(Q)$ through,

$$g(r) = 1 + \frac{1}{2\pi^2\rho_0 r}\int_0^\infty \left[S(Q) - 1\right]\sin(Qr)QdQ \, . \qquad \text{(S2)}$$

where $S(Q)$ is the structure function,

$$S(Q)=\frac{1}{4\pi Q^2 N}\sum_{j,j'}\left\langle e^{i\mathbf{Q}\cdot\left[r_j-r_{j'}\right]}\right\rangle, \quad \text{(S3)}$$

and $Q = |\mathbf{Q}|$. The $S_0(Q)$ of the ideal structure shown in Fig. 4 was calculated by

$$S_0(Q)=1+\frac{4\pi\rho_0}{Q}\int_0^{r_{Max}}\left[g_0(r)-1\right]\sin(Qr)W\left(\frac{r}{r_{Max}}\right)rdr\,. \quad \text{(S4)}$$

where r_{Max} is the termination point in r. $W(x)$ is the window function introduced to minimize termination error. We used the Lorch function, $W(x) = \sin y/y$, where $y = (5\pi/2)x$. The result shown in Fig. 4 was obtained with r_{Max} = 50 Å. The first peak becomes a δ-function for an infinite sample.

5. *First peak of S(Q)*

The first peak of $S(Q)$ for $Pd_{42.5}Ni_{7.5}Cu_{30}P_{20}$ is fit better by the Lorentzian function than the Gaussian function as shown in Fig. S4. We found that the first peak of $S(Q)$ for the alloys with relatively high values of $S(Q_1)$ has a Lorentzian shape, whereas that for the alloys with low values of $S(Q_1)$ it is more Gaussian, reflecting diversity in atomic sizes. For the liquids with the Gaussian shape $S(Q)$ deviates from eq. (2), but it is still possible to determine the structural coherence length, which is found to be nearly proportional to the first peak height of the total $S(Q) - 1$. This point will be discussed in further detail elsewhere.

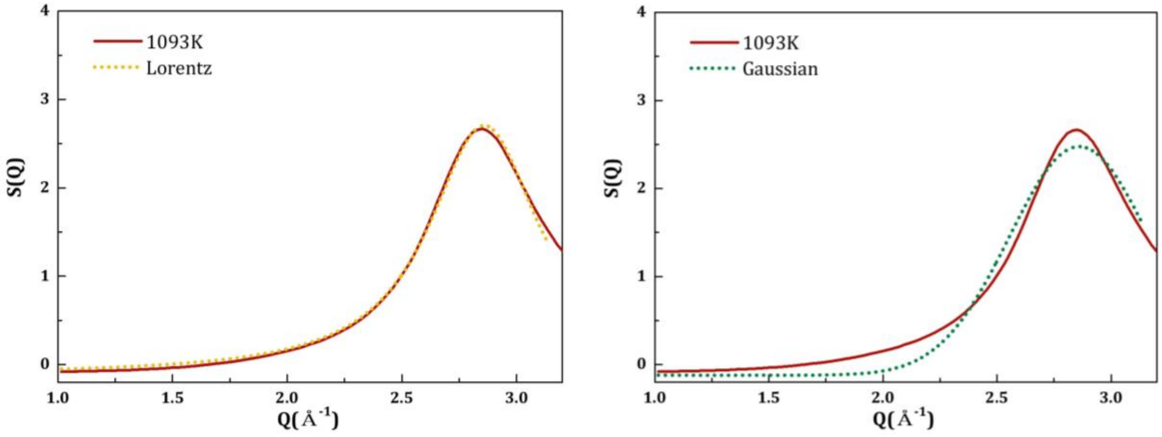

Fig. S4 Fit for the first peak of $S(Q)$ experimentally determined for $Pd_{42.5}Ni_{7.5}Cu_{30}P_{20}$ at 1093 K by Lorentzian (left) and by a Gaussian (right) functions.

6. *Relation to fragility*

The experimentally determined values of fragility, m, in eq. (6) [13, 51 – 55], were found to be related to the structural fragility, m_s, defined by eq. (5), by $m \propto m_S^c$, with c = 3.6, as shown in Fig. S5. Here the values of m_s determined by simulation are compared with the values of m determined by experiment. Therefore the difference between c (= 3.6) and d (= 3.0) in eq. (7) should not be considered significant. For the plot shown in Fig. 3 the activation energy for

viscosity is defined by $\eta(T) = \eta_\infty exp(E_a(T)/k_B T)$, with $\eta_\infty = h\rho$, where h is the Planck's constant and ρ is atomic number density [31, 56].

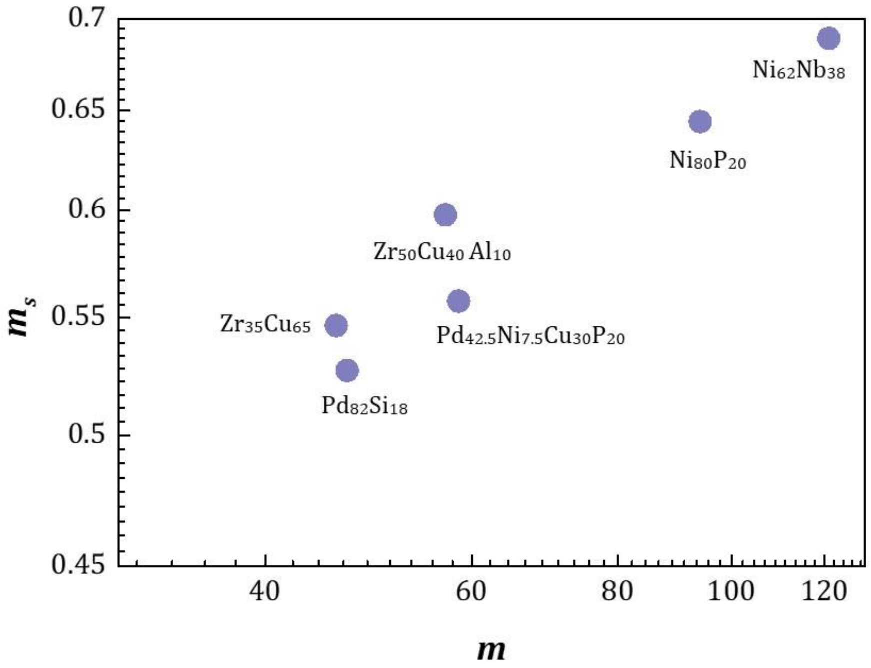

Fig. S5 The log-log plot of fragility, m, vs. structural fragility, m_s, for various alloys. The values of m are for $Pd_{42.5}Ni_{7.5}Cu_{30}P_{20}$ [13], $Pd_{82}Si_{18}$ [51], $Zr_{35}Cu_{65}$ [52], $Zr_{50}Cu_{40}Al_{10}$ [53], $Ni_{62}Nb_{38}$ [54], and $Ni_{80}P_{20}$ [55].

7. Pseudospin model of shear stress in liquid

The atomic-level stress is defined by

$$\sigma_i^{\alpha\beta} = \frac{1}{V_i}\sum_j f_{ij}\frac{r_{ij}^{\alpha}r_{ij}^{\beta}}{r_{ij}}, \tag{S5}$$

where α, β denote Cartesian coordinates, V_i is the local atomic volume of the i-th atom and f_{ij} is the two-body force between atoms i and j [57]. It was found that in supercooled liquid the atomic-level shear stresses develop spatial correlations [58]. A pseudospin model was developed for a two-dimensional model liquid to explain such correlations [25]. Focusing on a specific shear stress, σ_i^{xy}, a pseudospin, s_i, was defined as

$$s_i = \begin{cases} 1, & if \quad \sigma_i^{xy} > 0. \\ -1, & if \quad \sigma_i^{xy} < 0. \end{cases} \tag{S6}$$

The effective Hamiltonian,

$$H = -\sum_{i} \sum_{j}^{j \neq i, r_{ij} < r_c} J_2 \sin 2\theta_{ij} \left(s_i + s_j \right) s_i s_j \ , \qquad \text{(S7)}$$

where θ_{ij} is the angle $\boldsymbol{r_{ij}}$ makes with the x-axis. For an LJ liquid $J_2 = -2.5$, in the Lennard-Jones (LJ) unit, reproduces the stress correlation [25]. The same applies to the other shear stress component $(x^2 - y^2)$. Because of the anisotropy in the exchange it is not easy to locate the ground state. But it is clear that alignment along the same axis, for instance $x = y$ for $s_i = 1$, is strongly disfavored. If $s_i = 1$, s_j found in the direction of <11> ($x = y$) will be in the −1 state. Therefore the ferromagnetic state never develops. Given the frustration-prone structure, the ground state is most likely the spin-glass state.

8. The structure of the ideal liquid

Starting from the random structure the model was refined by the reverse Monte-Carlo method to fit to $G_0(r)$ shown in Fig. S6. To avoid atoms coming too close to each other a hard-sphere cut-off (the minimum distance constraint) was set at r_{co} (= 1 Å, 1.5 Å or 2.0 Å), but otherwise fitting was carried out without restriction. The $G(r)$ of the RMC generated models with varying r_{co} is shown in Fig. S7, indicating that the results are not strongly affected by the value of r_{co} except for the first peak. The model appears random (Fig. S8) even though it has long-range order. The Bragg sphere allows a high density of the three particle terms, $\rho_{Q1}\rho_{Q2}\rho_{-(Q1+Q2)}$, in the Landau expansion of the free energy [8], contributing to the stability of the ideal glass state.

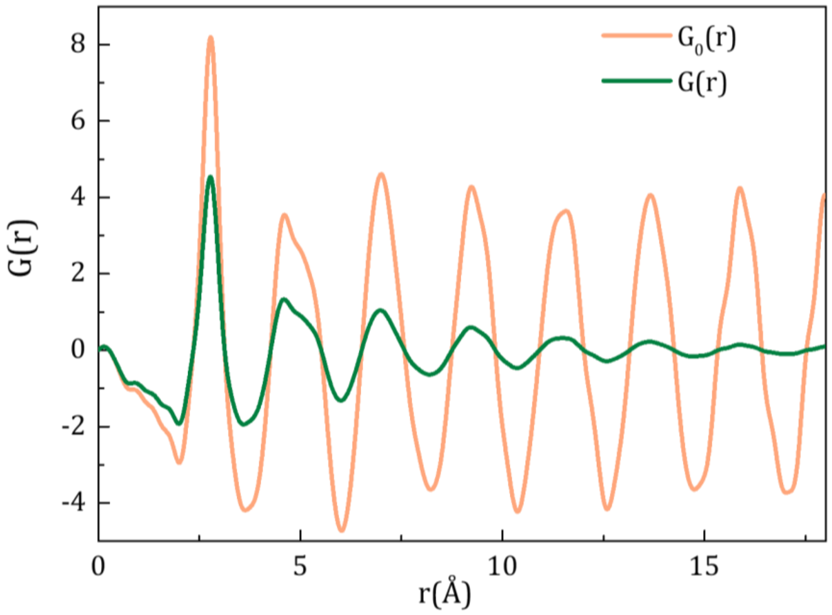

Fig. S6 The reduced PDF, $G(r)$, for $Pd_{42.5}Ni_{7.5}Cu_{30}P_{20}$ at 600 K (green) and $G_0(r)$ obtained by multiplying through $\exp(r/\xi_s)$ (orange).

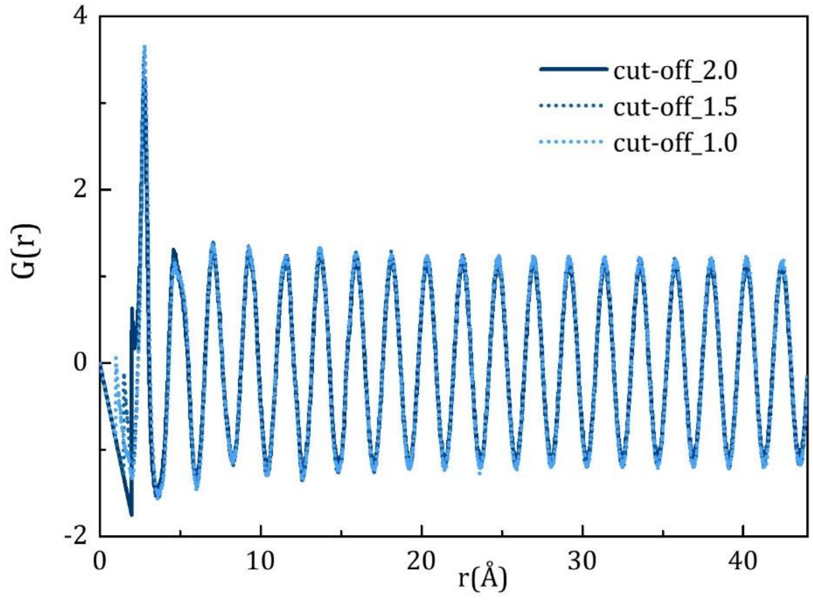

Fig. S7 $G(r)$ of the RMC generated ideal structure for cut-off values of 1.0, 1.5 and 2.0 Å. The long-range part of the structure is not affected by the choice of the cut-off value.

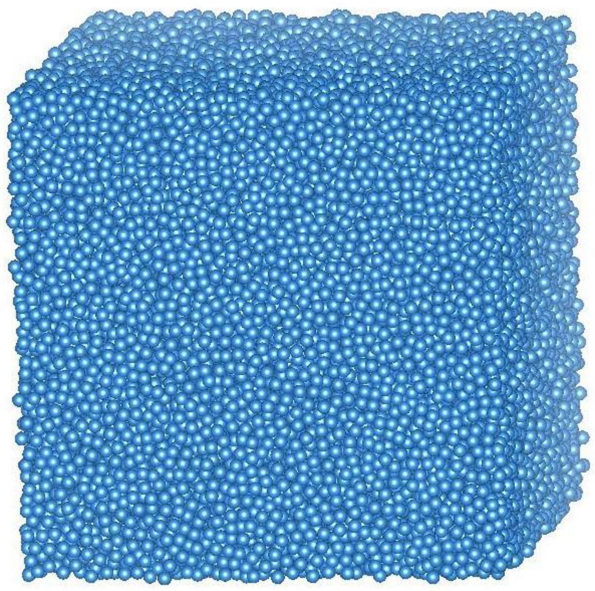

Fig. S8 A model of the ideal liquid generated by RMC simulation with r_{co} = 2 Å.

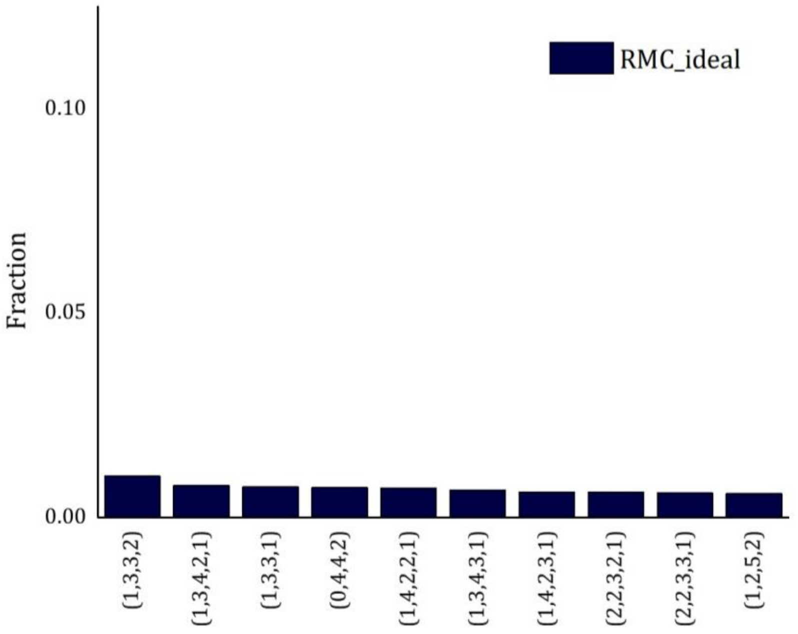

Fig. S9 Distribution of the fraction of the Voronoi polyhedra in the ideal liquid structure obtained by the RMC method with r_{co} = 2 Å. Here (n_3, n_4, n_5, n_6) expresses the number of Voronoi faces with triangular (n_3), square (n_4) faces, etc. None of the local structures dominate the distribution. The population of the icosahedral structure (0, 0, 12, 0) is 0.7%.

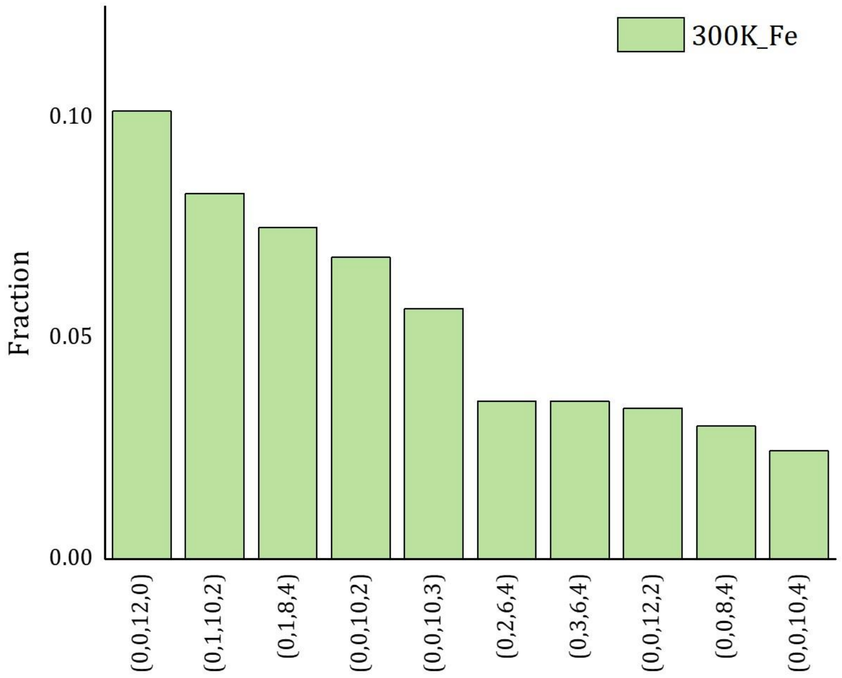

Fig. S10 Distribution of the Voronoi polyhedra in the model liquid Fe at 300K.

The amplitude of oscillation in $G(r)$ of the model (Fig. 5) is only about 1/3 of that for $G_0(r)$, because of the frustration between short- and long-range correlations which apparently modeling could not overcome. The model fits well with $G_1(r)$, the $G(r)$ at 600 K multiplied through $\exp((r - r_c)/\xi_s)$ for $r > r_c$, with $r_c = 3.67$ Å. The long-range correlation, however, is not obvious in the picture of the model (Fig. S8). The Voronoi analysis [16] of the ideal structure shows a very wide distribution of local structures as shown in Fig. S9. In comparison, that of the liquid Fe glass (Fig. S10) is dominated by a relatively small number of local structures, including the icosahedra (0, 0, 12, 0) and icosahedra-like structures, such as (0, 0, 10, 2).

9. Origin of the Curie-Weiss law

We summarize our argument for the Curie-Weiss law below. Further details are discussed in Ref. 36. In crystalline solids the thermal effect on the structure function $S(Q)$ is expressed by the Debye-Waller factor due to thermal vibration. But in liquids the structure is not fixed, and evolves with temperature. The variation in $S(Q_1) - 1$ reflects such structural evolution, rather than the effects of atomic vibration. The ideal glass structure with long-range order is described by the local density function,

$$\rho(\boldsymbol{r}) = \frac{1}{V}\int \rho(\boldsymbol{Q_1})\exp(i\boldsymbol{Q_1}\cdot\boldsymbol{r})\,d\Omega_1\,, \tag{S8}$$

where Ω_1 is the solid angle of $\boldsymbol{Q}_1$, and $\rho(\boldsymbol{Q}_1)$ is the density function in $\boldsymbol{Q}$ space which has a random phase factor to ensure even mass distribution. Now we fix the position of i-th atom, and introduce the displacement of its nearest neighbor, j-th atom, $\boldsymbol{u}_j$. Then,

$$\rho(\boldsymbol{r}_j) = \frac{1}{V}\int \rho(\boldsymbol{Q_1})\exp\left(i\boldsymbol{Q_1}\cdot\left(\boldsymbol{r}_j^0 + \boldsymbol{u}_j\right)\right)d\Omega_1\,. \tag{S9}$$

where $\boldsymbol{r}_j^0$ is the original position. The ensemble average for $\boldsymbol{u}_j$ is,

$$\left\langle \rho(\boldsymbol{r}_j)\right\rangle = \frac{1}{V}\int \rho(\boldsymbol{Q_1})\exp\left(i\boldsymbol{Q_1}\cdot\boldsymbol{r}_j^0\right)\exp\left(\left\langle i\boldsymbol{Q_1}\cdot\boldsymbol{u}_j\right\rangle\right)d\Omega_1 = \rho\left(\boldsymbol{r}_j^0\right)\exp\left(-\frac{1}{2}Q_1^2\left\langle u_j^2\right\rangle\right) \tag{S10}$$

Therefor the medium-range density correlation between atoms i and j is reduced by the factor,

$$F = \exp\left(-\frac{Q_1^2\left\langle u^2\right\rangle}{2}\right) = \exp\left(-\frac{a}{\xi_s}\right), \tag{S11}$$

where a is the atomic distance. Thus the structural coherence length, ξ_s, is given by

$$\xi_s = \frac{2a}{Q_1^2\left\langle u^2\right\rangle} = \frac{2}{Q_1^2 a\left\langle \varepsilon^2\right\rangle}\,. \tag{S12}$$

where $\varepsilon = u/a$ is the atomic-level strain. As the origin of ε, we use the atomic-level stress, eq. (S5), which describes the distortion in the nearest neighbor cage [35, 57]. The atomic-level stress tensors have six components, but because $\boldsymbol{f}_{ij} = -\boldsymbol{f}_{ji}$, they have $3N$ degrees of freedom. They can replace the atomic coordinates as the thermodynamic variables, and satisfy the equipartition theorem. Thus ε^2 is proportional to temperature T [58, 59],

$$\varepsilon^2 = \frac{2}{Q_1^2 a \xi_s} = \left(5 + \frac{G}{3B}\right)\frac{k_B T}{6VG}. \quad \text{(S13)}$$

where B and G are the bulk and shear moduli, and V is the atomic volume. Thus the height of the first peak of $S(Q)$ is,

$$S(Q_1,T) - 1 = \frac{\xi(T)}{\pi} F = \frac{2}{\pi Q_1^2 a \varepsilon^2} F, \quad F = \frac{2\pi^2 \rho_0 N_C}{Q_1^2} \quad . \quad \text{(S14)}$$

$$\frac{1}{S(Q_1,T) - 1} = \frac{\pi Q_1^2 a k_B T_g}{12FVG}\left(5 + \frac{G}{3B}\right)\frac{T}{T_g} = C\frac{T}{T_g}. \quad \text{(S15)}$$

where N_C is the coordination number. So far we assumed that the fluctuations in the nearest neighbors are uncorrelated. This assumption is reasonable for liquids at high temperatures, but when a cage is formed by definition neighbors are correlated. The atomic-level stress of the central atom is reduced by the elastic relaxation of the cage and the matrix beyond. This effect can be described by the Eshelby theory of elastic inclusion [35]. In the Eshelby theory [60] a spherical elastic object is strained by the transformation strain, ε^{T} so that it fits the hole in the elastic medium. Then the elastic medium is relaxed to accommodate the inclusion. The total strain is given by,

$$\varepsilon^2 = \frac{\left(\varepsilon^T\right)^2}{K_\gamma} \quad . \quad \text{(S16)}$$

where

$$K_\gamma = \frac{15(1-\nu)}{7-5\nu}, \quad \text{(S17)}$$

for shear strain and ν is the Poisson's ratio. The total strain satisfies the equipartition theorem, thus $\varepsilon^2 \propto kT$. However, $S(Q)$ reflects ε^{T}, because it is determined by the atomic positions which reflects the topology of medium-range atomic connectivity. Therefore,

$$S(Q) - 1 \propto \frac{1}{\left(\varepsilon^T\right)^2} \propto \frac{1}{K_\gamma(T) kT} \quad , \quad \text{(S18)}$$

where $K_\gamma(T)$ is the temperature dependent Eshelby constant. At $T \to \infty$ it is equal to unity, and upon cooling it gradually increases to the value in eq. (S17) at low temperatures. In a simple binary model of liquid-like (uncaged) and solid-like (caged) regions [31, 61] accommodation occurs only in the solid-like regions. Thus,

$$\frac{1}{K_\gamma(T)} = 1 - \left(1 - \frac{1}{K_\gamma}\right) f(T) \quad \text{(S19)}$$

and $f(T)$ is the volume fraction of the caged atoms, or the solid-like atoms. If we assume $f(T) = \alpha \tilde{S}(T)$, $\tilde{S}(Q_1,T) = S(Q_1,T) - 1$, we obtain the Curie-Weiss law,

$$\tilde{S}(Q_1,T) = S(Q_1) - 1 = \frac{T_g/C}{T - T_{IG}}. \tag{S20}$$

where

$$\frac{T_{IG}}{T_g} = \left(1 - \frac{1}{K_\gamma}\right)\frac{\alpha}{C}. \tag{S21}$$

References:

49. Sachdev, S. & Nelson, D. R. Statistical mechanics of pentagonal and icosahedral order in dense liquids, *Phys. Rev. B* **32,** 1480-1502 (1985).
50. Leheny, R. L., Menon, N., Nagel, S. R., Price, D. L., Suzuya, K. & Thiyagarajan, P. Structural studies of an organic liquid through the glass transition. *J. Chem. Phys.* **105,** 7783-7794 (1996).
51. Chen, N., Li, Y. & Yao, K. F. Thermal stability and fragility of Pd–Si binary bulk metallic glasses. *J. Alloys & Compounds* ***504S*,** S211–S214 (2010).
52. Jaiswal, A., Egami, T., Kelton, K. F., Schweizer, K. S. & Zhang, Y. Correlation between Fragility and the Arrhenius Crossover Phenomenon in Metallic, Molecular, and Network Liquids. *Phys. Rev. Lett.* 117, 205701 (2016).
53. Qiao, J.C., Casalini, R. & Pelletier, J. M. Main (α) relaxation and excess wing in Zr50Cu40Al10 bulk metallic glass investigated by mechanical spectroscopy. *J. Non-cryst. Solids* **407**, 106-109 (2015).
54. Komatsu, T. Application of fragility concept to metallic glass formers. *J. Non-cryst. Solids* **185**, 199-202 (1995).
55. Johnson, W. L., Na, J. H. & Demetriou, M. D. Quantifying the origin of metallic glass formation. *Nature Comm.* **7**, 10313 (2016).
56. Eyring, H. Viscosity, plasticity, and diffusion as examples of absolute reaction rates. *J. Chem. Phys.* **4,** 283 (1936).
57. Egami, T., Maeda, K. & Vitek, V. Structural defects in amorphous solids A computer simulation study. *Phil. Mag. A* **41,** 883-901 (1980).
58. Chen, S.-P., Egami, T. & Vitek, V. Local fluctuations and ordering in liquid and amorphous metals. *Phys. Rev. B* **37,** 2440-2449 (1988).
59. Levashov, V. A., Aga, R. S., Morris, J. R. & Egami, T. Equipartition theorem and the dynamics of liquids. *Phys. Rev. B*, **78,** 064205 (2008).
60. Eshelby, J. D. The determination of the elastic field of an ellipsoidal inclusion, and related problems. *Proc. Roy. Soc. London A* **241,** 376-396 (1957).
61. Cohen, M. H. & Grest, G. Liquid-glass transition, a free-volume approach. *Phys. Rev. B* **20,** 1077-1098 (1979).
